# Artificial intelligence in head and neck cancer diagnosis

**DOI:** 10.1016/j.jpi.2022.100153

**Published:** 2022-11-08

**Authors:** Sara Bassani, Nicola Santonicco, Albino Eccher, Aldo Scarpa, Matteo Vianini, Matteo Brunelli, Nicola Bisi, Riccardo Nocini, Luca Sacchetto, Enrico Munari, Liron Pantanowitz, Ilaria Girolami, Gabriele Molteni

**Affiliations:** a Otolaryngology-Head and Neck Surgery Department, University of Verona, Verona, Italy; b Department of Diagnostics and Public Health, Section of Pathology, University and Hospital Trust of Verona, Verona, Italy; c Department of Pathology and Diagnostics, University and Hospital Trust of Verona, Verona, Italy; d Department of Otolaryngology, Villafranca Hospital, Verona, Italy; e Department of Molecular and Translational Medicine, University of Brescia, 25121 Brescia, Italy; f Department of Pathology, University of Michigan, Ann Arbor, MI, USA; g Department of Pathology, Provincial Hospital of Bolzano (SABES-ASDAA), Bolzano-Bozen, Italy; Lehrkrankenhaus der Paracelsus Medizinischen Privatuniversität

**Keywords:** Artificial intelligence, Head and neck cancer, Pathology, Diagnosis, Whole slide imaging

## Abstract

**Introduction:**

Artificial intelligence (AI) is currently being used to augment histopathological diagnostics in pathology. This systematic review aims to evaluate the evolution of these AI-based diagnostic techniques for diagnosing head and neck neoplasms.

**Materials and methods:**

Articles regarding the use of AI for head and neck pathology published from 1982 until March 2022 were evaluated based on a search strategy determined by a multidisciplinary team of pathologists and otolaryngologists. Data from eligible articles were summarized according to author, year of publication, country, study population, tumor details, study results, and limitations.

**Results:**

Thirteen articles were included according to inclusion criteria. The selected studies were published between 2012 and March 1, 2022. Most of these studies concern the diagnosis of oral cancer; in particular, 6 are related to the oral cavity, 2 to the larynx, 1 to the salivary glands, and 4 to head and neck squamous cell carcinoma not otherwise specified (NOS). As for the type of diagnostics considered, 12 concerned histopathology and 1 cytology.

**Discussion:**

Starting from the pathological examination, artificial intelligence tools are an excellent solution for implementing diagnosis capability. Nevertheless, today the unavailability of large training datasets is a main issue that needs to be overcome to realize the true potential.

## Introduction

Head and neck squamous cell carcinoma (HNSCC) affects approximately 880 000 new patients each year worldwide and represents a leading cause of mortality in some countries.^1^ The main risk factors of HNSCC are tobacco smoking, alcohol abuse. and human papillomavirus (HPV) infection.^2^ The 5-year overall survival is globally 50%–65% at 5 years with combined therapeutic strategy (surgery alone, surgery combined with adjuvant treatment, and exclusive radiotherapy with or without chemotherapy).^2^ In general, HNC is often diagnosed at advanced stages, and prognosis depends on anatomic site of involvement, stage, and HPV status.^3^ Primary and secondary prevention are key points in HNC management. Diagnosis usually requires an otolaryngological examination and eventually an endoscopic evaluation when required. Pre-treatment evaluations include radiological examinations and histopathological analysis of procured tissue.^4^ However, these diagnostic methods are usually dependent upon the interpretation of human pathologists,^5^^,^^6^ which can result in inconsistencies in cancer diagnosis, grading, and prognostication.^7^^,^^8^ Diagnostic error can negatively impact patient outcome.

The field of pathology has witnessed significant developments in recent years. From a biological standpoint is the turning point in the therapy of HNSCC that ensued with the introduction of immunotherapy.^9^^,^^10^ From a technological standpoint is the widespread adoption of whole slide imaging (WSI) which has advanced diagnosis and research. Accruement of large digital datasets from scanning pathology glass slides has boosted to the the application of AI in pathology. By using AI-based tools to analyze histopathology whole slide images, we are now able to enhance and possibly automate pathology diagnosis, as well as better interrogate and quantify parameters of the tumor microenvironment. AI algorithms developed using deep learning methods are based on the concept of an artificial neural network trained using a large number of digital images to subsequently classify unknown images. In general, as the volume of training data increases the AI model performance improves.^11^ This learning process can be "supervised" by utilizing human experts to annotate histopathological images, or it can be "unsupervised" by training the algorithm to deduce data directly from preset information. AI-based tools in pathology have the potential to automate workflow processes, improve diagnosis, standardize reporting, quantify scoring, and provide information that most pathologists today are unable to provide using light microscopy such as reporting about prognosis and predicting therapy response.^12^

The aim of this article is to provide an overview of the existing scientific literature on the applications of AI for the pathological diagnosis of HNC.

## Materials and methods

**Fig. 1:**
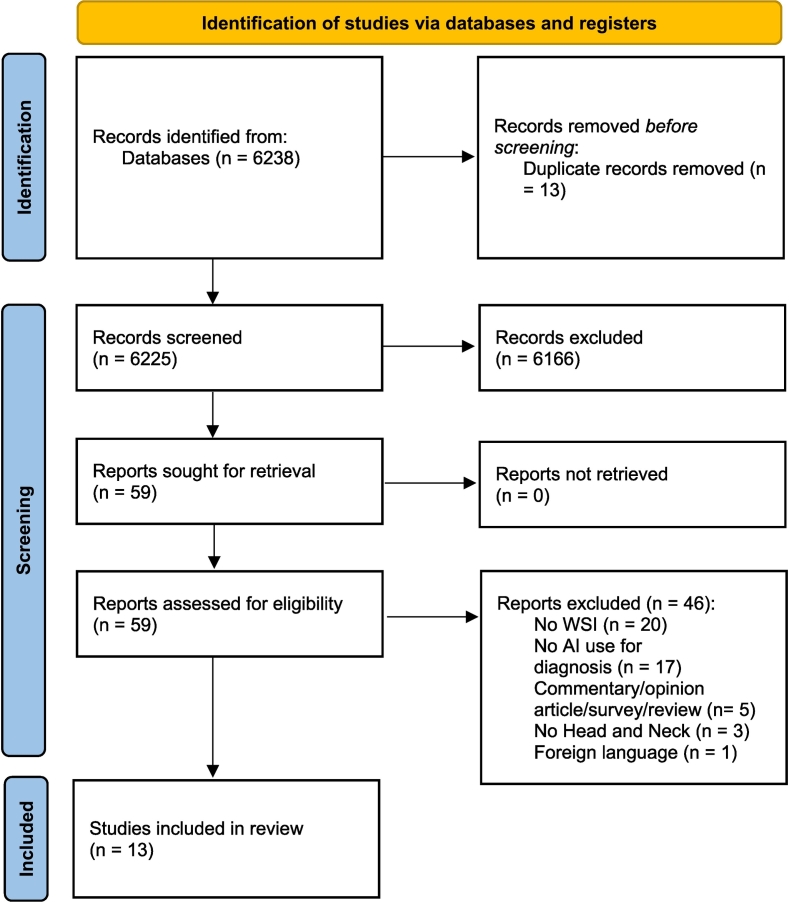
PRISMA Flow diagram: flowchart of the systematic review according to the PRISMA guidelines.

This study aimed to comply with the Preferred Reporting Items for Systematic reviews and Meta-Analysis (PRISMA) guidelines.^13^ A systematic search was carried out in the electronic databases Pubmed-MEDLINE and Embase including all archived literature until March 1, 2022. The complete search string for Pubmed-MEDLINE and Embase is shown in Table S1 of the supplementary material. Two authors (SB, NS) independently reviewed all article titles and abstracts with the aid of Rayyan QCRI reference manager web application.^14^ Full texts of the articles that fulfilled the initial screening criteria were acquired and reviewed for subsequent inclusion against the eligibility criteria (Fig. 1). Any disagreement with respect to inclusion of a particular article was resolved by consensus. In the second stage of study selection, the same authors independently assessed full-text reports to obtain a list of relevant articles. Inclusion criteria were as follows:

• Studies using AI for histopathological and/or cytological diagnosis (detection, grading. and classification) of malignant HNC.
• Studies evaluating diagnostic accuracy of AI and ML algorithms for malignant HNC.
• Studies evaluating accuracy of AI and ML algorithms in the differential diagnosis of precancerous and malignant HNC lesions.

Exclusion criteria:

• Studies not using WSI.
• Studies not using pathology imaging modalities (e.g., radiology and endoscopy images).
• Studies regarding AI/ML not primarily investigating the detection, grading, and classification of HNC (e.g., those predicting progression, prognosis, or treatment efficacy).
• Studies using AI/ML for only thyroid, skin, and esophageal cancer detection.
• Studies using AI/ML for only benign head and neck lesions.
• Narrative reviews, letters to editors, commentaries, and conference abstracts.
• Studies not available in the English language.

Data from eligible articles were summarized in a standard format that included: authors, year of publication, country, type of study, aim, study population, primary tumor location, type of diagnosis, main study results, and limitations (Table 2). The primary outcome was to assess the histopathological features used for diagnosis and grading of the head and neck lesion under study, in addition to the methods and performance of the proposed AI/ML algorithms. A descriptive analysis was subsequently conducted, and the performance of the algorithms used, when present, was reported.

## Results

Of 6225 abstracts evaluated, 13 articles satisfied inclusion criteria and were accordingly included. The flow chart depicting article screening is shown in Fig. 1. The selected studies were published between 2012 and March 1, 2022. Most of these studies concern the diagnosis of oral cancer; in particular, 6 are related to the oral cavity, 2 to the larynx, 1 to the salivary glands, and 4 to HNSCCC not otherwise specified (NOS). As for the type of diagnostics employed, 12 concerned histopathology and 1 cytology. The AI algorithms tested were: U-Net (a convoluted neural network architecture), Support Vector Machine (SVM) (a supervised machine learning algorithm), InceptionV3 (a deep convolutional neural network), Chan-Vese algorithm (an active contour model), Decision Tree, Linear discriminant (a supervised machine learning algorithm), K-Nearest Neighbor (a supervised machine learning algorithm), InceptionV4 (a convoluted neural network architecture), GoogLe Net (a convoluted neural network architecture), MobilNet-v2 (a convoluted neural network architecture), ResNet50 (a convoluted neural network architecture), ResNet101 (a convoluted neural network architecture), Random Forest (a supervised machine learning algorithm), Gaussian naive Bayes (a supervised machine learning algorithm), Logistic regression (a supervised machine learning algorithm), Tree Model (a supervised machine learning algorithm).

### Oral cavity

Six papers were about oral cavity HNC. Macaulay et al. evaluated the use of AI for the cytological recognition of suspicious lesions. Cytology samples from 369 patients were included and different algorithms were compared, with the best accuracy of 92.5% achieved in recognizing normal samples and 89.4% in recognizing suspicious ones.^15^ Das et al. introduced an AI-based tool for diagnosing oral squamous cell carcinoma (OSCC) based on identification of keratinized areas and keratin pearls. Their study population consisted of 10 patients diagnosed with OSCC from a single institution. Thirty digitized images were used as ground truth and the Chan-Vese method was used as a diagnostic algorithm validated by 2 expert pathologists. The Chan-Vese method is an algorithm designed to segment objects without clearly defined edges.^16^ The average performance of this segmentation method was evaluated by the Jaccard coefficient (77.76%), yielding a first-rate correlation value (0.85) and segmentation accuracy (95.08%).^17^ Another study by Das et al. described a convolutional neural network (CNN) used to distinguish epithelial, subepithelial, and keratinizing regions as well as detect keratin pearls in OSCC. CNN is an artificial neural network that contains many layers to perform operations through numerous hyperparameters, that can be useful to build a classification model. CNNs permit to learn different high-level features from the set of image patches at different layers and testing then into classifiers. This model is inspired from the animal visual cortex structure. Images from 25 patients with low-grade OSCC, 15 with high-grade, and 2 healthy subjects from a single center were evaluated. The performance of their deep learning algorithm for epithelial layer segmentation was as follows: accuracy 98.42%, sensitivity 97.76%, Jaccard coefficient 90.63%, dice coefficient 95.03%; to distinguish the keratin region the performance was: accuracy 96.88%, Jaccard coefficient 71.87%, dice coefficient 75.19%; and the performance for keratin pearl detection was: accuracy 96.88%.^18^ In the study by Rahman et al., images (n = 134) with normal tissue and images (n = 135) with malignancy from a multicentric case series were used. These authors’ model achieved 89.7% accuracy for the dataset generated by applying t-test and 100% accuracy for the principal component analysis (PCA) generated datasets. T-test and PCA are 2 different methods used to select significant features; PCA, in particular, is statistical data compression technique used to identify a smaller number of uncorrelated variables. Area under the curve (AUC) was 0.92 and 1.0 for the first and second datasets.^19^ In another paper by Rahman et al., performance of 5 classifiers was further evaluated for the recognition of OSCC based on characteristics such as shape, texture, and color, obtaining an accuracy, specificity, sensitivity, and precision of over 99% with four classifiers except one (K-Nearest Neighbor classifier).^20^ Dos Santos et al. tested a CNN model to determine OSCC areas within oral mucosal samples. Fifteen WSIs and 1050 image patches extracted from a single center were evaluated. Their proposed model showed an accuracy of 97.6%, precision of 91.1%, Dice coefficient of 92.0%, Jaccard coefficient of 85.2%, specificity of 98.4%, and sensitivity of 92.9%.^21^

### Larynx

Two studies dealing with the larynx were included. Zhou et al. developed a dual-modality optical imaging microscope combined with machine learning algorithms for the automatic detection of laryngeal squamous cell carcinoma (SCC). Image patches derived from 20 slides, from 10 patients, from a single center were used. Algorithms used were SVM, Random Forest, Gaussian naive Bayes, and Logistic regression. The highest accuracy was reached using SVM.^22^ In the study by He et al., a method for diagnosing laryngeal SCC was tested. A pool of 3458 pathological images were taken from 1228 patients underwent AI-aided endoscopy of the upper aerodigestive tract to look for suspicious laryngeal lesions that were biopsied and histologically examined. The pathology images were randomly divided into a training dataset, a validation dataset, and a testing dataset. The algorithm used was Inception V3 and AUC was 0.994 for the validation dataset and 0.981 for the testing dataset.^23^

### HNSCC NOS

Four studies dealing with HNSCC where included. Halicek et al. used a CNN on 381 WSIs from 156 patients to diagnose HNSCC, both on the primary tumor and at the lymph node level. Results in terms of performance were: accuracy 84.8 ± 1.6%, sensitivity 84.7 ± 2.2%, specificity 85.0 ± 2.2%.^24^ Mavuduru et al. evaluated the ability of a CNN using U-Net tested on 200 tissue samples from 84 HNSCC patients from a single center. Their study showed an AUC of the testing group of 0.89, with a threshold of 0.2845 and average time of segmentation from WSI to be 72 s.^25^ In the study of Rodner et al., a CNN was used to diagnose HNSCC distinguishing between cancer, normal epithelium, background stroma, and other tissue types on 114 images from 12 patients. Average and overall recognition rates for the 4 classes were 88.9% and 86.7%, respectively. Average segmentation time was 113 s, where the best segmentation time was 55 s.^26^ In the study by Tang et al., a CNN was used to extract high-dimensional features from hematoxylin and eosin slides to detect HNCSCC in 135 cervical lymph nodes from 20 patients. In their primary model that used all 4 CNNs, the accuracy was between 97.3% and 98.7%, and the AUC was between 0.9957 and 0.9982. In general, performance of the development dataset were 100% for accuracy, sensitivity, and specificity; whereas in the test dataset sensitivity was 100%, specificity 75.9%, and accuracy 86%.^27^ Only 1 study regarded salivary glands tumors. Lopez-Janeiro et al. used a tree model algorithm to diagnose different histotypes of malignant tumors on 115 samples of salivary glands. The total accuracy achieved was 84.6%.^28^

## Discussion

Digital pathology has evolved from using static images to the WSI era. Despite technical and diagnostic issues,^29^ WSI has shown great diagnostic concordance compared to traditional light microscopy in anatomical pathology,^30^, ^31^, ^32^ including cytopathology.^33^ WSI has enabled the application of AI tools in pathology.^34^^,^^35^ A brief and general description of different AI modalities is summarized in Table 1.

**Table 1:** Brief description of the different modalities.

Artificial intelligence	Computer science branch aiming to build smart machines to perform tasks
Machine learning	Ability for machines to learn information and patterns from data
Supervised learning	Training machines from labelled input and output data
Unsupervised learning	Training machines by extracting hidden patterns from input and output that have not been labelled
Whole slide image	High-resolution microscopic digital image
Deep learning	Sub-field of machine learning in which algorithms learn without supervision

**Table 2:** Details of the studies included.

Author, year, country	Type of study and aim	Study population	Primary tumor location	Type of diagnostic	Algorithm used	Main results	Main limits
Das et al., 2015; India	Automated identification of keratinization and keratin pearl area from in situ oral histological images	10 patients	Oral cavity	Histology	Chan-Vese algorithm	Accuracy: 95.08%. Correlation value: 0.85. Jaccard coefficient: 77.76%	Small study population, single center, only SCC
Das et al., 2018; India	CNN to distinguish epithelial, subepithelial and keratin region and to detect keratin pearls in OSCCC	25 low grades SCC, 15 high grades SCC and two healthy patients	Oral cavity	Histology	CNN	Epithelial layer segmentation: accuracy 98.42%, sensitivity 97.76%, Jaccard coefficient 90.63%, Dice coefficient 95.03%. Keratin region segmentation: accuracy 96.88%, Jaccard coefficient 71.87%, Dice coefficient 75.19%. Keratin pearls detection: accuracy 96.88%	Small study population, single center, only SCC
Dos Santos et al., 2021; Brazil	Detection of tumor regions from oral cavity tissue samples	15 WSI (1050 image-patches extracted from them)	Oral cavity	Histology	CNN	Accuracy: 97.6%. Precision: 91.1%. Dice coefficient: 92.0%. Jaccard coefficient: 85.2%. Specificity: 98.4%. Sensitivity: 92.9%.	Small study population, single center, only SCC
Halicek et al, 2019; USA	Investigate the ability of CNNs for detecting head and neck SCC (primary and lymph-nodes) and thyroid carcinoma using WSI	381 WSI from 156 patients	Head and neck and thyroid	Histology	CNN	SCC detection: accuracy 84.8%, sensitivity 84%, specificity 85%. Thyroid: accuracy 89.4%, sensitivity 89.6%, specificity 89.1%. Lymph-node: accuracy 93.4%, sensitivity 93.6%	Single center, only SCC
He et al., 2021; China	Diagnosis of laryngeal SCC by AI. Phase 1: AI-assisted assessment of suspicious areas in NBI light. Phase 2: biopsy of suspicious areas and pathological analysis with the aid of AI.	3458 pathological images (752 benign and 2,706 LSCC) of 1228 patients	Larynx	Histology	InceptionV3	AUC for pathology group data: 0.994 for the validation data set and 0.981 for the testing data set.	Only SCC
Lopez-Janeiro et al., 2022; Spain	A machine learning algorithm to approach the diagnosis of malignant salivary gland tumors (12 variables)	115 samples	Salivary glands	Histology	Tree model	Accuracy: 84.6%.	Single center
Macaulay et al., 2012; Canada	Image cytometry for detection of suspicious lesions in the oral cavity.	369 cytological samples from 369 patients (148 samples from pathology-proven sites of SCC, carcinoma in situ or severe dysplasia; 77 samples from sites with inflammation, infection, or trauma, and 144 samples from normal sites)	Oral cavity	Cytology	Set of discriminate functions	Best algorithm accuracy: 92.5% (normal samples), 89.4% (abnormal samples).	Single center, only SCC
Mavuduru et al., 2020; USA	Evaluate ability of full CNN U-Net to perform segmentation of SCC	200 tissue samples from 84 HNSCC patients	Head and neck	Histology	U-Net	Validation dataset: accuracy 74%, sensitivity 79%, specificity 68%. Testing dataset: accuracy 82%, sensitivity 81%, specificity 82%.	Single center, only SCC
Rahman et al., 2017; India	Textural pattern classification for oral squamous cell carcinoma	134 images with normal tissue and 135 images with malignant tissue	Oral cavity	Histology	SMV	Accuracy for data set by applying t-test: 89.7%. Accuracy for the PCA generated data sets: 100%. AUC: 0.92 (first data set) and 1 (second data set).	Small study population, bicentric study, only SCC
Rahman et al., 2020; India	Automated oral squamous cell carcinoma identification using shape, texture and color features of whole image strips.	42 whole slide slices	Oral cavity	Histology	5 classifiers: Decision Tree, Support Vector Machine, Linear discriminant, K-Nearest Neighbor, Logistic regression	Best results with SVM and logistic regression: sensitivity 100%, precision 100%, specificity 100%, accuracy 100%. Worst performance with K-nearest Neighbor: sensitivity 99.2%, precision 36.7%, specificity 16.1%, accuracy 43.5%.	Small study population, bicentric study, only SCC
Rodner et al., 2018; Germany	Fully convolutional networks in multimodal nonlinear microscopy images for automated detection of head and neck carcinoma detecting four classes: cancer, normal epithelium, background, and other tissue types.	114 images and 12 patients from patients with HNSCC (1 oral cavity; 5 oropharynges; 4 larynx; 2 hypopharynx)	Head and neck	Histology	Fully convolutional neural network (FCN)	Average recognition rate: 88.9% Overall recognition rate or the four classes: 86.7%.	Single center, only SCC
Tang et al., 2022; China	Use a deep learning method to extract high dimensional features from H&E slides to detect tumor in HNSCC lymph nodes.	135 lymph nodes slides from 20 patients	Head and neck	Histology	CNN (GoogLe Net, MobilNet-v2, ResNet50, ResNet101)	Development data set: 100% of accuracy, sensitivity and specificity. Test data set: sensitivity 100%, specificity 75,9%, accuracy 86%.	Small study population, single center, only SCC
Zhou et al., 2021; USA	A dual-modality optical imaging microscope combining hyperspectral imaging and polarized light imaging and incorporating polarized hyperspectral imaging with machine learn algorithms for automatic detection of SCC.	4500 image patches of 20 slides from 10 patients	Larynx	Histology	SVM, Random forest, Gaussian naive Bayes, Logistic regression	Best accuracy reached with SMV: 92%, 92,9%, 80,3% and 93,5% for each vector parameter.	Single center, only SCC

AI: artificial intelligence, AUC: area under the curve, CNN: convolutional neural network, H&E: hematoxylin and eosin; HNSCC: head and neck squamous cell carcinoma, LSCC: laryngeal squamous cell carcinoma, NBI: narrow band imaging, OSCC: oral squamous cell carcinoma, SCC: squamous cell carcinoma, SVM: Support vector machine, WSI: whole slide image

Our review included 13 studies. Tumor site varied among these included papers, reflecting the complexity of the head and neck region. Despite various primary anatomical sites of involvement, the predominant histotype was SCC in nearly all of the papers, except for Lopez-Janeiro et al. who studied salivary gland tumors. All studies were conducted on images of histology, except for MacAulay et al. who used 369 cytological samples from oral cavity brushings. Slides were scanned using Cyto-Savant, which is an automated quantitative system used largely for cervical cytology and sputum samples, with software that automatically segments and focuses all objects on the slide. This system showed good accuracy to correctly recognize normal and abnormal oral cavity samples. Further application of AI tools in cytology samples is anticipated.

Regarding the performance of AI tools in histology, all studies reported an accuracy over 90%, except for 2 papers.^24^^,^^27^ They also reported excellent performance via AUC calculations, as well as high sensitivity and specificity. Different types of AI algorithms were tested in the included studies, from supervised machine learning systems (e.g., SVM) to deep learning systems such as CNNs. While deep learning is more advanced than simple machine learning algorithms, this AI methodology introduces new challenges. With deep learning larger datasets are typically required for training to develop an accurately performing model. For this reason, if training datasets are limited or of poor quality (e.g., lack heterogeneity), deep learning performance could be hindered. Of note, the quantity of data used in the included studies was restricted and imperfect. In this review, both supervised and unsupervised models showed good performances. All papers included were focused on SCC, with the exception of the study by Lopez-Janeiro et al. that was focused on salivary gland tumors. Salivary gland neoplasms are a heterogeneous group of tumors, and given the fact that they represent a diagnostic challenge for pathologists due to overlapping morphologic features further AI-assisted diagnostic tools in this area would be applauded.

## Disclosure

The authors declare that they have no known competing financial interests or personal relationships that could have appeared to influence the work reported in this paper.

## Appendix A Supplementary data

Supplementary material Image 1
