# Supplementary material for: Artificial intelligence in head and neck cancer diagnosis
Source: J Pathol Inform. 2022 Nov 8;13:100153. doi: 10.1016/j.jpi.2022.100153 (PMC9808017; doi:10.1016/j.jpi.2022.100153)
Supplement: Supplementary file 1 — Supplementary material [file mmc1.docx]

| **Database** | **Search key** |
| --- | --- |
| *Pubmed* |  |
|  | #1 "image"[Title/Abstract] AND "analysis"[Title/Abstract]  #2 "artificial"[Title/Abstract] AND "intelligence"[Title/Abstract]  #3 "morphometry"[Title/Abstract] OR "morphometric"[Title/Abstract] OR "histomorphometric"[Title/Abstract] OR "AI"[Title/Abstract] OR "algorithm*"[Title/Abstract] OR "neural network"[Title/Abstract] OR "neural networks"[Title/Abstract] OR "convolutional"[Title/Abstract] OR "deep-learning"[Title/Abstract] OR "deep-learning"[Title/Abstract] OR "computational"[Title/Abstract] OR "computerized"[Title/Abstract] OR "automated"[Title/Abstract] OR "machine-learning"[Title/Abstract] OR "machine-learning"[Title/Abstract]  #4 #1 OR #2 OR #3  #5 "head"[Title/Abstract] AND "neck"[Title/Abstract]  #6 "HNSCC"[Title/Abstract] OR "squamous"[Title/Abstract] OR "salivary"[Title/Abstract] OR "mucoepidermoid"[Title/Abstract] OR "adenoid"[Title/Abstract] OR "adenoma"[Title/Abstract] OR "secretory"[Title/Abstract] OR "duct"[Title/Abstract] OR "acinic"[Title/Abstract] OR "polymorphous"[Title/Abstract] OR "myoepithelial"[Title/Abstract] OR "lymphoepithelial"[Title/Abstract] OR "odontogenic"[Title/Abstract]  #7 #5 OR #6  #8 "pathology"[Title/Abstract] OR "histological"[Title/Abstract] OR "histology"[Title/Abstract] OR "cytology"[Title/Abstract] OR "cytological"[Title/Abstract] OR "whole-slide"[Title/Abstract] OR "whole-slide"[Title/Abstract] OR "WSI"[Title/Abstract] OR "digital"[Title/Abstract]  #9 #4 AND #7 AND #8 |
| *Embase* |  |
|  | #1 ('image'/exp OR image) AND ('analysis'/exp OR analysis)  #2 artificial AND ('intelligence'/exp OR intelligence)  #3 morphometry OR morphometric OR histomorphometric OR ai OR algorithm* OR 'neural network' OR 'neural networks' OR convolutional OR 'deep learning' OR computational OR computerized OR automated OR 'machine learning'  #4 #1 OR #2 OR #3  #5 head AND neck  #6 HNSCC OR squamous OR salivary OR mucoepidermoid OR adenoid OR adenoma OR secretory OR duct OR acinic OR polymorphous OR myoepithelial OR lymphoepithelial OR odontogenic OR premalignant OR precancerous OR preneoplastic OR intraepithelial OR “carcinoma in situ” OR dysplasia OR dysplastic  #7 #5 AND #6  #8 pathology OR histological OR histology OR cytology OR cytological OR smear OR cell-block OR whole-slide OR “whole slide” OR WSI OR digital  #9 #4 AND #7 AND #8  #10 AND [embase]/lim NOT ([embase]/lim AND [medline]/lim)  #15 AND [embase]/lim NOT ([embase]/lim AND [medline]/lim) AND ('article'/it OR 'article in press'/it OR 'conference paper'/it OR 'review'/it) AND ('cancer cell culture'/de OR 'cancer model'/de OR 'case control study'/de OR 'case report'/de OR 'clinical article'/de OR 'clinical protocol'/de OR 'clinical trial'/de OR 'cohort analysis'/de OR 'comparative study'/de OR 'computer model'/de OR 'computer simulation'/de OR 'controlled clinical trial'/de OR 'controlled study'/de OR 'correlational study'/de OR 'cross sectional study'/de OR 'diagnostic test accuracy study'/de OR 'ex vivo study'/de OR 'feasibility study'/de OR 'human'/de OR 'human cell'/de OR 'human experiment'/de OR 'human tissue'/de OR 'in vivo study'/de OR 'intermethod comparison'/de OR 'major clinical study'/de OR 'mathematical model'/de OR 'medical record review'/de OR 'meta analysis'/de OR 'multicenter study'/de OR 'normal human'/de OR 'observational study'/de OR 'pilot study'/de OR 'practice guideline'/de OR 'prospective study'/de OR 'quality control'/de OR 'questionnaire'/de OR 'randomized controlled trial'/de OR 'randomized controlled trial topic'/de OR 'retrospective study'/de OR 'sample size'/de OR 'simulation'/de OR 'systematic review'/de OR 'validation process'/de OR 'validation study'/de) |
